# High-flow nasal cannula oxygen versus non-invasive ventilation in patients with acute hypoxaemic respiratory failure undergoing flexible bronchoscopy - a prospective randomised trial

**DOI:** 10.1186/s13054-014-0712-9

**Published:** 2014-12-22

**Authors:** Marcel Simon, Stephan Braune, Daniel Frings, Ann-Kathrin Wiontzek, Hans Klose, Stefan Kluge

**Affiliations:** Department of Intensive Care Medicine, University Medical Centre Hamburg-Eppendorf, Martinistr. 52, 20246 Hamburg, Germany; Department of Respiratory Medicine, University Medical Centre Hamburg-Eppendorf, Martinistr. 52, 20246 Hamburg, Germany

## Abstract

**Introduction:**

Critically ill patients with respiratory failure undergoing bronchoscopy have an increased risk of hypoxaemia-related complications. Previous studies have shown that in awake, hypoxaemic patients non-invasive ventilation (NIV) is helpful in preventing gas exchange deterioration during bronchoscopy. An alternative and increasingly used means of oxygen delivery is its application via high-flow nasal cannula (HFNC). This study was conducted to compare HFNC with NIV in patients with acute hypoxaemic respiratory failure undergoing flexible bronchoscopy.

**Methods:**

Prospective randomised trial randomising 40 critically ill patients with hypoxaemic respiratory failure to receive either NIV or HFNC during bronchoscopy in the intensive care unit.

**Results:**

After the initiation of NIV and HFNC, oxygen levels were significantly higher in the NIV group compared to the HFNC group. Two patients were unable to proceed to bronchoscopy after the institution of HFNC due to progressive hypoxaemia. During bronchoscopy, one patient on HFNC deteriorated due to intravenous sedation requiring non-invasive ventilatory support. Bronchoscopy was well tolerated in all other patients. There were no significant differences between the two groups regarding heart rate, mean arterial pressure and respiratory rate. Three patients in the NIV group and one patient in the HFNC group were intubated within 24 hours after the end of bronchoscopy (*P* = 0.29).

**Conclusions:**

The application of NIV was superior to HFNC with regard to oxygenation before, during and after bronchoscopy in patients with moderate to severe hypoxaemia. In patients with stable oxygenation under HFNC, subsequent bronchoscopy was well tolerated.

**Trial registration:**

ClinicalTrials.gov NCT01870765. Registered 30 May 2013.

## Introduction

Flexible bronchoscopy (FB) is a frequently performed procedure for the assessment, diagnosis, and treatment of patients with respiratory disease in the intensive care unit (ICU). The procedure and applications of FB have progressively evolved and expanded since it was first introduced in 1968 and it is now well established as an integral diagnostic and therapeutic tool in respiratory and critical care medicine [1,2].

While bronchoscopy is generally considered a safe procedure [3], it is well known that critically ill patients undergoing bronchoscopy are at an increased risk for complications, most of all the deterioration of pre-existing hypoxaemia [4].

Few randomised controlled studies and case series have shown that the use of continuous positive airway pressure (CPAP) or non-invasive ventilation (NIV) is superior to conventional means of oxygen delivery in patients with hypoxaemia in terms of preventing deterioration of gas exchange during bronchoscopy [5-12].

High-flow nasal cannula (HFNC) oxygen utilises higher gas flow rates than conventional low-flow oxygen systems. Oxygenation via HFNC is increasingly applied in adult ICU patients with acute hypoxaemic respiratory failure as an alternative to NIV [13]. The devices used deliver heated and humidified oxygen at a flow of up to 60 litres per minute via nasal cannulas. This results in effective and sustained improvement in respiratory parameters in patients with acute hypoxaemic respiratory failure by several mechanisms [14].

In a pilot study, Lucangelo *et al*. found that HFNC improves oxygenation in patients undergoing bronchoscopy [15]. However, the patients investigated were not hypoxaemic and there was no comparison with NIV. Therefore, we conducted this prospective randomised trial comparing HFNC with NIV in patients with acute hypoxaemic respiratory failure undergoing FB to assess the ability to maintain oxygen saturation during bronchoscopy as well as changes in blood gases and outcome following bronchoscopy.

## Methods

### Study design

The study was conducted as a prospective randomised trial. All patients admitted to the Department of Intensive Care Medicine at the University Medical Centre Hamburg-Eppendorf were eligible for study inclusion. Prior to enrolment, all participants or their legal representatives gave written informed consent. The study was approved by the ethics committee of the chamber of physicians in Hamburg, Germany, and the trial was registered at ClinicalTrials.gov (registration number NCT01870765, registration date 30 May 2013).

### Study population

Medical and surgical patients treated in one of the ten departmental ICUs were enrolled. Inclusion criteria were (1) respiratory failure with hypoxaemia defined as partial pressure of oxygen in arterial blood/fraction of inspired oxygen (PaO_2_/FiO_2_) below 300 mm Hg, (2) indication for diagnostic and/or therapeutic FB, (3) age 18 years or above and (4) informed consent. Exclusion criteria were (1) contraindications for NIV or HFNC, (2) nasopharyngeal obstruction or blockage, (3) indication for intubation and (4) pre-existing invasive ventilation.

Simplified acute physiology scores II (SAPS II) were calculated according to standard criteria [16]. Immunosuppression was defined as a neutrophil count of less than 1,000/mL, immunosuppressive medication, chemotherapy within the last 60 days or acquired immunodeficiency syndrome.

### Study protocol

After enrolment, patients were randomised to receive either NIV or HFNC. Randomisation was accomplished by computer-generated random number sequence and the allocation sequence was concealed from the study team enrolling and assessing participants by using numbered, opaque and sealed envelopes.

**Figure 1:**
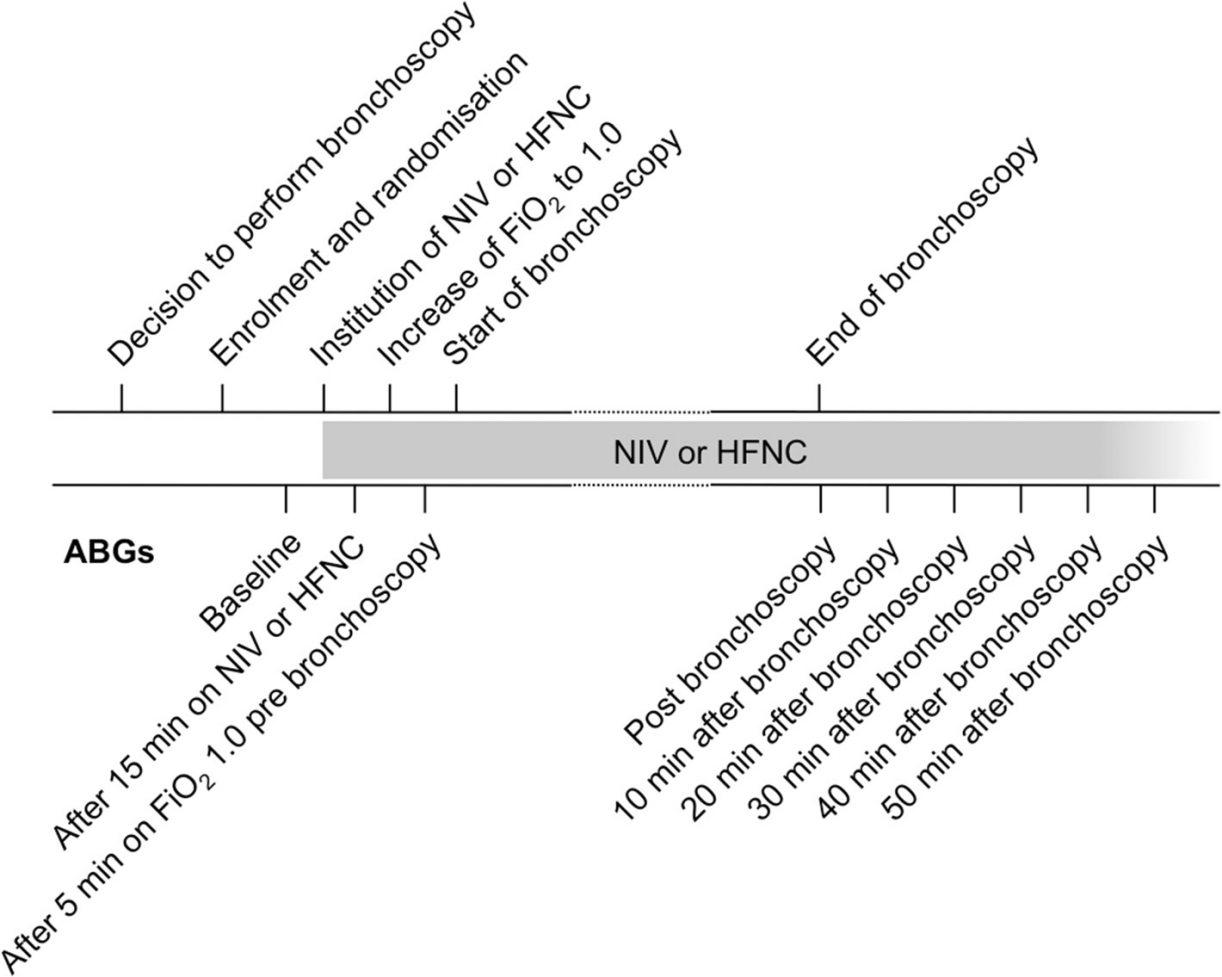
**Study workflow.** Abbreviations: ABG, arterial blood gas analysis; FiO_2_, fraction of inspired oxygen; HFNC, high-flow nasal cannula; NIV, non-invasive ventilation.

Arterial blood gases were drawn from a catheter in the radial or femoral artery at baseline, 15 minutes after the institution of NIV or HFNC, after 5 minutes on FiO_2_ 1.0 just before the start of bronchoscopy, at the end of bronchoscopy as well as 10, 20, 30, 40 and 50 minutes after the completion of bronchoscopy. Blood pressure, heart rate, respiratory rate and oxygen saturation recorded by pulse oximetry (SpO_2_) were monitored constantly throughout this period. For details on study workflow see the flow diagram in Figure 1.

### Non-invasive ventilation (NIV)

NIV was administered using ICU ventilators in NIV mode (Carina™ or Evita Infinity V500™, Dräger, Germany). A full face mask (Medisize, Neunkirchen-Seelscheid, Germany) secured with elastic banding was used as the interface. A swivel connector was inserted between the mask and ventilator tubing to allow for the insertion of the bronchoscope. The ventilator mode was set to pressure support mode or pressure controlled mode. Positive end-expiratory pressure (PEEP) was set between 3 and 10 cm H_2_O and inspiratory pressures between 15 and 20 cm H_2_O to achieve adequate oxygenation and ventilation. The adjustment of ventilator settings was left to the discretion of the treating intensivist.

### High-flow nasal cannula (HFNC) oxygen

To deliver high-flow oxygen, an Optiflow™ system with a medium-size adult nasal cannula as patient interface (Fisher and Paykel Healthcare Ltd, Auckland, New Zealand) was used in all cases. The oxygen flow was set to 50 litres per minute.

### Flexible bronchoscopy (FB)

**Figure 2:**
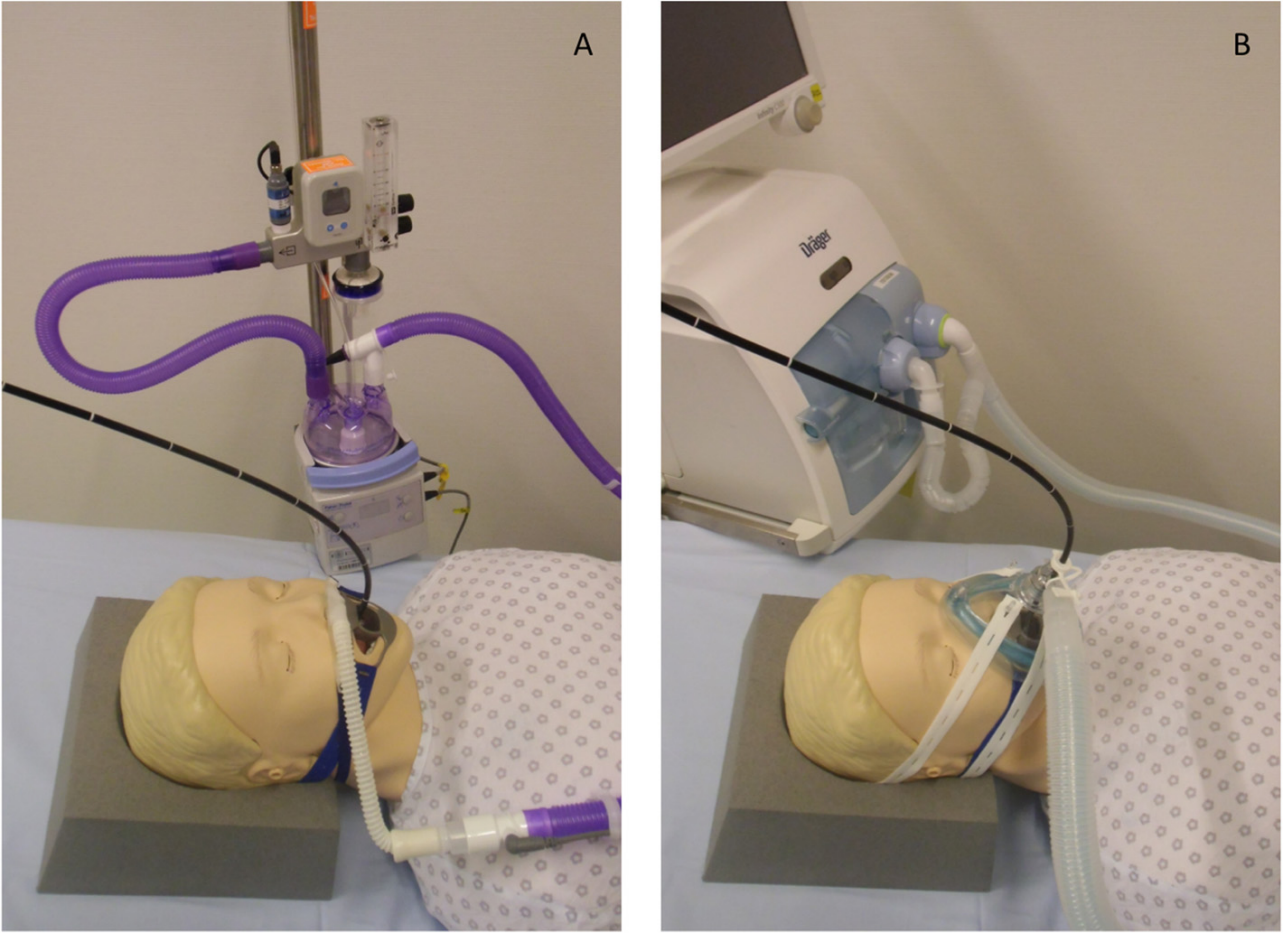
**Illustration of bronchoscopy using HFNC (A) or NIV (B).** HFNC, high-flow nasal cannula; NIV, non-invasive ventilation.

All bronchoscopies were performed by experienced pulmonologists. The decision to perform bronchoscopy was not part of the study and was left to the discretion of the treating intensivist. The FiO_2_ was increased to 1.0 prior to the start of bronchoscopy and adjusted to maintain an arterial oxygen saturation of more than 90% after the completion of bronchoscopy. A flexible bronchoscope (BF-P60™, Olympus, Tokyo, Japan) passed through the mouth was used for all procedures. The setup using NIV or HFNC is illustrated in Figure 2. Intravenous sedation was achieved in a standardised manner using repetitive bolus applications of 10 to 20 mg of propofol every 2 to 3 minutes. Topical anaesthesia was applied to the nasal and pharyngeal mucosa using lidocain gel and spray and to the tracheobronchial mucosa using 5 mL of lidocain 0.8% applied through the working channel of the bronchoscope in aliquots of 1 mL. After inspection of the tracheobronchial tree, the bronchoscope was wedged in the appropriate subsegmental bronchus. Bronchoalveolar lavage (BAL) was performed using normal saline being instilled in aliquots of 20 mL and then aspirated. The number and type of diagnostic tests ordered determined the amount of fluid required. Depending on the underlying condition, BAL fluid was sent for cytological or microbiological analyses. The duration of bronchoscopy was defined as the time between insertion and removal of the bronchoscope from the tracheobronchial tree.

### Outcome parameters

Primary outcome parameter was the lowest oxygen saturation recorded by pulse oximetry during FB. Secondary outcome parameters were (1) changes in blood gases for up to 50 minutes after the procedure and (2) the requirement for intubation within 8 hours of completion of FB and at any other point during ICU stay. Intubation was considered a complication possibly related to bronchoscopy, if it occurred within 8 hours of the procedure. This time frame was adopted from previous studies [7,17,18]. The decision to intubate was left to the discretion of the treating intensivist in accordance with published guidelines [19]. Sample size was calculated to allow the detection of a 3% difference in minimal oxygen saturation during FB assuming an alpha risk of 0.05 and a power of 0.8.

### Statistical analysis

Results are presented as absolute numbers and percentages, as mean and standard deviation for continuous data if normally distributed and as median and range if not normally distributed. Comparison between the two groups was performed using the *t* test or the Mann-Whitney *U* test for metric data and the chi-square test for categorical data. A two-sided *P* value of less than 0.05 was considered to be significant. The software used for descriptive analyses was SPSS (version 20.0, SPSS Inc., Chicago, IL, USA).

## Results

### Patient characteristics

Between July 2013 and December 2013, 44 patients met inclusion criteria and were found eligible to participate in the study. Of these, two patients declined informed consent. Two patients who were randomised to receive HFNC were unable to proceed to bronchoscopy after the institution of HFNC due to progressive hypoxaemia requiring the initiation of NIV: one patient who had previously been breathing spontaneously on HFNC deteriorated due to the retention of secretions, the other patient who had been on NIV deteriorated when transferred to HFNC. Eventually, 40 patients were enrolled in the study and were randomised to undergo FB while on HFNC or on NIV.

Mean PaO_2_/FiO_2_ at baseline was 138 ± 69 mm Hg in the HFNC group and 163 ± 64 mm Hg in the NIV group (*P* = 0.25). Mean partial pressure of carbon dioxide in arterial blood (PaCO_2_) levels at baseline were significantly higher in the NIV group (43 ± 13 mm Hg) than in the HFNC group (34 ± 6 mm Hg) (*P* = 0.01). Table 1 provides further details on patient characteristics.

**Table 1:** **Patient characteristics**

	**NIV**	**HFNC**	***P*** **value**
Total number of patients	20	20	
Gender			
Male	13 (65%)	11 (55%)	0.52
Female	7 (35%)	9 (45%)	
Age (years)	68 ± 11	64 ± 12	0.28
SAPS II score	46 ± 10	43 ± 13	0.39
Thrombocytopenia (<50 Mrd/L)	3 (15%)	5 (25%)	0.43
Immunosuppression	8 (40%)	5 (25%)	0.31
Use of vasopressors	10 (50%)	10 (50%)	1.00
Antibiotic therapy	17 (85%)	19 (95%)	0.29
Antimycotic therapy	5 (25%)	6 (30%)	0.72
Antiviral therapy	3 (15%)	7 (35%)	0.14
Main diagnosis			
Haematological disorder	4 (20%)	7 (35%)	0.29
Sepsis	4 (20%)	3 (15%)	0.68
Lung cancer	2 (10%)	3 (15%)	0.63
Extrapulmonary solid cancer	2 (10%)	3 (15%)	0.63
Liver cirrhosis	1 (5%)	1 (5%)	1.00
Trauma	1 (5%)	1 (5%)	1.00
Interstitial lung disease	1 (5%)	1 (5%)	1.00
Alveolar haemorrhage	1 (5%)	0	0.31
Community-acquired pneumonia	1 (5%)	0	0.31
Chronic obstructive pulmonary disease	1 (5%)	0	0.31
Pulmonary arterial hypertension	1 (5%)	0	0.31
Acquired immunodeficiency syndrome	0	1 (5%)	0.31
Ileum perforation	1 (5%)	0	0.31
Indication for bronchoscopy			
Hospital-acquired pneumonia	10 (50%)	14 (70%)	0.50
Community-acquired pneumonia	5 (25%)	3 (15%)	
Suspected retention of secretions	3 (15%)	2 (10%)	
Suspected interstitial lung disease	1 (5%)	0	
Suspected alveolar haemorrhage	1 (5%)	0	
Suspected malignancy	0	1 (5%)	
Therapy at baseline			
Low-flow oxygen via nasal cannula	5 (25%)	2 (10%)	0.23
Low-flow oxygen via face mask	1 (5%)	3 (15%)	
HFNC	9 (45%)	13 (65%)	
NIV	5 (25%)	2 (10%)	
Physiological parameters at baseline			
Heart rate (beats/min)	95 ± 14	101 ± 15	0.27
Mean arterial pressure (mm Hg)	85 ± 11	82 ± 14	0.56
Respiratory rate (breaths/min)	30 ± 8	30 ± 9	0.86
PaO_2_/FiO_2_ (mm Hg)	163 ± 64	138 ± 69	0.25
PaCO_2_ (mm Hg)	43 ± 13	34 ± 6	0.01
pH	7.43 ± 0.11	7.46 ± 0.07	0.21

Values are given as mean and standard deviation or as numbers and percentages. FiO_2_, fraction of inspired oxygen; HFNC, high-flow nasal cannula; NIV, non-invasive ventilation; PaCO_2_, partial pressure of carbon dioxide in arterial blood; PaO_2_, partial pressure of oxygen in arterial blood; SAPS II, simplified acute physiology score II.

### Tolerance of the procedure

**Figure 3:**
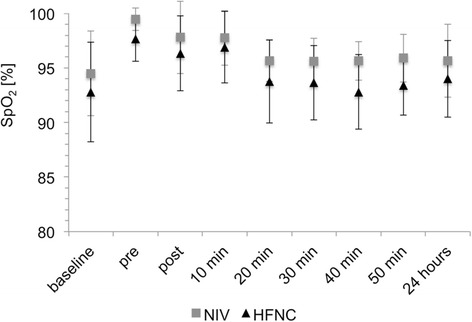
**SpO**_**2**_ **at baseline, pre and post bronchoscopy.** Changes in SpO_2_. Values are given as mean and standard deviation. HFNC, high-flow nasal cannula; NIV, non-invasive ventilation; SpO_2_, oxygen saturation recorded by pulse oximetry.

The lowest SpO_2_ during bronchoscopy was 95 ± 5% in the NIV group and 92 ± 7% in the HFNC group (*P* = 0.07). At the pre-defined time points during the 50 minutes of follow-up as well as at 24 hours after the completion of bronchoscopy, no SpO_2_ of less than 85% was registered in any of the two groups. SpO_2_ values before and after bronchoscopy are shown in Figure 3.

**Figure 4:**
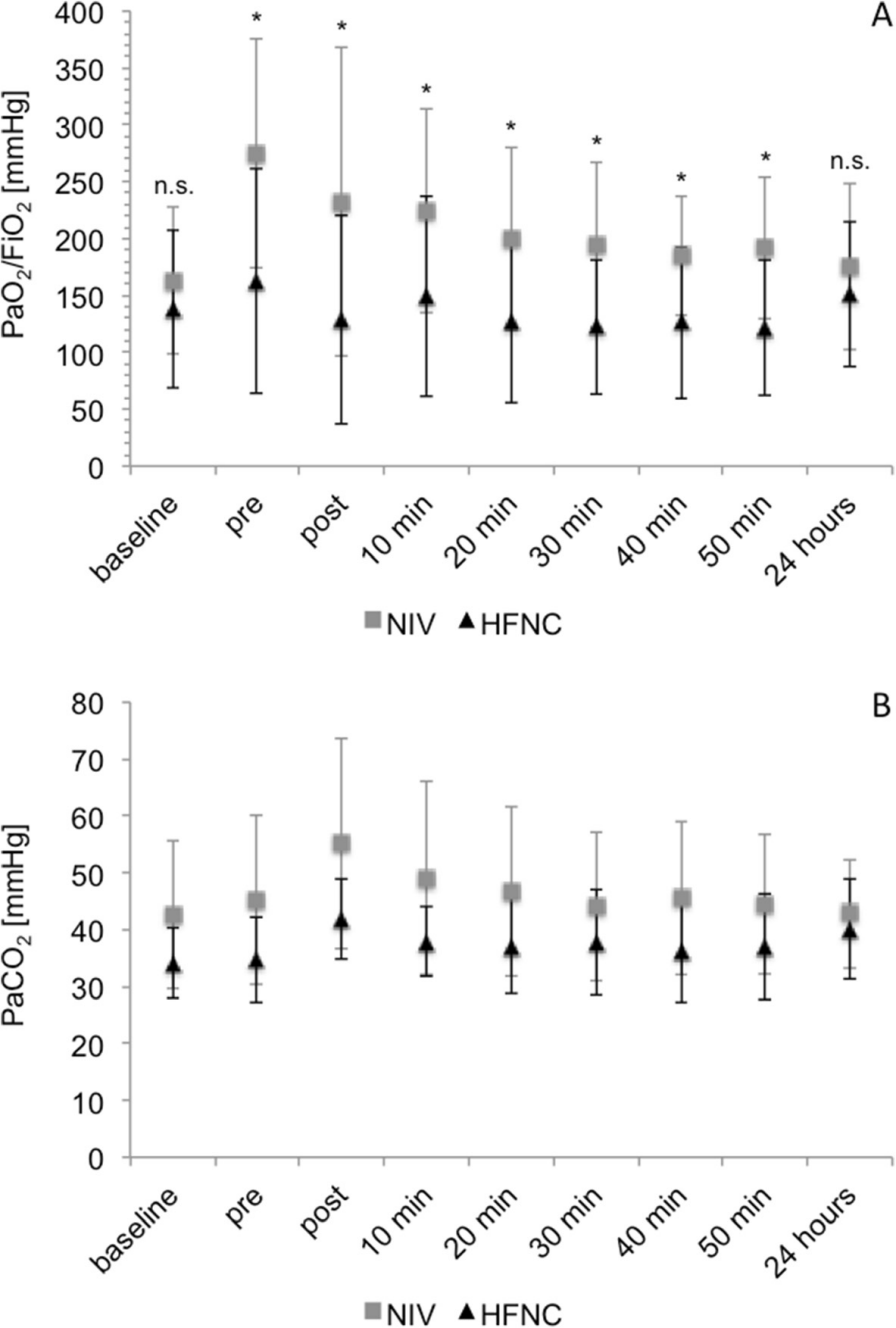
**PaO**_**2**_ **/FiO**_**2**_ **and PaCO**_**2**_ **at baseline, pre and post bronchoscopy.** Changes in PaO_2_/FiO_2_ **(A)** and PaCO_2_ **(B)**. Values are given as mean and standard deviation. ^*^ *P* <0.05; n.s. not significant. FiO_2_, fraction of inspired oxygen; HFNC, high-flow nasal cannula; NIV, non-invasive ventilation; PaCO_2_, partial pressure of carbon dioxide in arterial blood; PaO_2_, partial pressure of oxygen in arterial blood.

A significant increase in PaO_2_/FiO_2_ after 15 minutes on NIV compared to baseline (*P* = 0.04) was observed in the NIV group, while there was no significant change in PaO_2_/FiO_2_ in the HFNC group (*P* = 0.96). Comparing the two groups after 15 minutes on NIV or HFNC, PaO_2_/FiO_2_ was significantly better in the NIV group (*P* = 0.002). This difference in oxygenation was preserved throughout bronchoscopy and during the 50 minutes of follow-up. There were no significant differences between the two groups concerning respiratory rates and the course of PaCO_2_ values. At 24 hours after the completion of bronchoscopy, there was no significant difference between the two groups concerning PaO_2_/FiO_2_ (*P* = 0.29) or any of the other recorded parameters. Figure 4 shows PaO_2_/FiO_2_ and PaCO_2_ before and after bronchoscopy.

One patient in the HFNC group deteriorated after the application of intravenous sedation resulting in apnea while on HFNC requiring transitioning to NIV. Subsequent bronchoscopy was completed without further incident. Bronchoscopy was well tolerated by all other patients. The average duration of bronchoscopy was 5.0 ± 2.7 minutes in the HFNC group and 5.5 ± 1.9 minutes in the NIV group (*P* = 0.56). Concerning the total amount of propofol used for sedation, there was no significant difference between the NIV group (74 ± 36 mg) and the HFNC group (96 ± 59 mg) (*P* = 0.24). A BAL was performed in all cases. The amount of BAL fluid instilled was not significantly different between the two groups (*P* = 0.92).

There were no significant differences between the two groups at any of the pre-specified time points with regard to heart rate and mean arterial pressure.

Patients who were on low-flow oxygen therapy at baseline and were randomised to undergo bronchoscopy under NIV, stayed on NIV for a median duration of 20 minutes (range 0 to 9 hours) after the end of bronchoscopy, while patients who were randomised to undergo bronchoscopy under HFNC, stayed on HFNC for a median duration of 7.8 hours (range 1.5 to 103 hours) after the end of bronchoscopy (*P* = 0.04).

### Outcome

One patient in the HFNC group required intubation immediately after the completion of bronchoscopy. He had exhibited the lowest PaO_2_/FiO_2_ value (64 mm Hg) of all patients at baseline. None of the other patients in the two groups required intubation within the pre-defined period of 8 hours after the end of bronchoscopy (*P* = 0.31). During the following course of their ICU stay, 13 patients (65%) in the NIV group and 9 patients (45%) in the HFNC group required intubation (*P* = 0.20). Overall, 3 patients in the NIV group and 1 patient in the HFNC group were intubated within 24 hours after the end of bronchoscopy (*P* = 0.29). On average, intubation was performed 59 hours (range 9 to 391 hours) after the completion of bronchoscopy in the NIV group and 75 hours (range 0 to 338 hours) in the HFNC group (*P* = 0.54). In all these cases, intubation was due to progression of the underlying disease and not a consequence of the bronchoscopic procedure. Twenty-eight-day mortality was 40% in the NIV group and 65% in the HFNC group (*P* = 0.11).

## Discussion

In this randomised study, NIV was superior to HFNC with regard to maintaining adequate oxygenation before, during and after bronchoscopy in patients with acute hypoxaemic respiratory failure. In two patients transition to HFNC was impossible due to progressive hypoxaemia. FB was well tolerated and was not associated with complications.

To our knowledge, this is the first study comparing HFNC with NIV in hypoxaemic patients undergoing FB. Only two previous reports have assessed the use of HFNC for bronchoscopy. Lucangelo *et al*. compared three groups of patients with mild hypoxaemia undergoing bronchoscopy while being on different types of oxygen supplementation [15]. At the end of bronchoscopy, oxygenation was significantly better in patients receiving HFNC with a high flow rate of 60 litres per minute compared with HFNC with a lower flow rate of 40 litres per minute or oxygen administration through a Venturi mask. Lomas *et al*. reported the case of a patient with myasthenia gravis and severe acute respiratory failure due to bilateral atelectasis who underwent successful bronchoscopy using HFNC [20].

The concept of high-flow oxygen originates from the treatment of premature infants as an alternative to nasal CPAP [21,22]. Due to the ease of application, simplicity and good patient tolerance, HFNC is increasingly used in adult patients with acute hypoxaemic respiratory failure as an alternative to NIV [13]. It has also been shown that HFNC is better tolerated and more comfortable than oxygen applied via face mask [23]. In addition, the use of this technique has been described as means for pre-oxygenation before intubation [24], in the post-extubation period [25], in patients with do-not-intubate order [26], in the palliative care setting [27], in the emergency department [28] and in patients with heart failure [29]. Most of these observational studies showed beneficial effects of HFNC compared with conventional oxygen therapy in terms of improved symptoms (dyspnea score), respiratory rate and oxygenation. In one of the few randomised studies, the authors compared HFNC with standard low-flow oxygen therapy in patients with mild to moderate hypoxaemic respiratory failure. Significantly more HFNC patients succeeded with their allocated therapy and also had significantly fewer desaturations [30]. However, in relation to its widespread use, there is still little evidence concerning the risks and benefits of this new technology. Moreover, most studies are lacking clinical outcome data and assessment of long-term effects [31].

A number of physiological effects of HFNC have been described. These include the washout of pharyngeal dead space, reduction of airway resistance, increase in end-expiratory lung volume and generation of positive airway pressure [13,14,31]. In our study, after the initiation of NIV or HFNC, oxygenation was significantly better in the NIV group than in the HFNC group (*P* = 0.002). This can, at least in part, be explained by higher positive airway pressures generated by NIV [32]. Positive airway pressures with HFNC have been documented. In line with other researchers, Parke *et al*. found a positive linear relationship between oxygen flow and airway pressure. In the study on ICU patients, a significant difference in the degree of positive airway pressure was seen dependent on whether the person’s mouth was open or closed: a mean positive airway pressure of 2.7 cm H_2_O was reported with the mouth closed, while it was only 1.2 cm H_2_O with the mouth open [33]. Accordingly, in a study on healthy volunteers, a median positive airway pressure of 7.4 cm H_2_O was observed with the mouth closed, while it was 2.7 cm H_2_O with the mouth open [34]. Patients with more severe acute respiratory distress may have more inconsistent airway pressures due to their breathing through an open mouth. A fundamental difference between HFNC and NIV is the fact that HFNC systems maintain a fixed flow and generate variable pressures, whereas many NIV systems generate a fixed pressure by utilising variable flow [13]. In contrast to the NIV group, where the average PEEP was 5.4 cm H_2_O, it can be assumed that no relevant PEEP levels were achieved in the HFNC group due to the procedure-related open mouth. This is most likely the main reason for the differences observed in oxygenation between the two groups.

In addition to hypoxaemia, bronchoscopy can be associated with hypercapnia and side effects of sedation. This happened in one case in our study where the need for NIV was due to intravenous sedation. A recent multi-centre study investigating 169 bronchoscopies in spontaneously breathing hypoxaemic patients showed that one-third of cases were complicated by an increased need for ventilatory support [12]. Thus, in patients with ventilatory failure (primary ventilatory pump failure or secondary due to sedation) support or maintenance of ventilation by NIV seems advantageous in comparison to HFNC.

The following methodological limitations need to be considered: first, despite randomisation baseline PaCO_2_ levels were higher in the NIV group. However, subsequent changes in PaCO_2_ over time were similar in both groups. Second, our results are only applicable to populations with similar characteristics. It is therefore important to note that most critically ill patients in this study had moderate to severe hypoxaemic respiratory failure, high SAPS II values and vasopressor support.

Although oxygenation levels were lower in the HFNC group, all patients who were stable on HFNC for 15 minutes tolerated subsequent bronchoscopy well. A significant difference concerning the need for intubation between the two groups could not be detected. However, this study was not powered to answer this question and further studies including more patients are needed to assess this topic. Thus, patients stable on HFNC may undergo bronchoscopy without the need for transitioning to NIV. This may facilitate the procedure for ICU staff and provide improved patient comfort due to the avoidance of the potentially unpleasant experience of NIV. The fact that patients on low-flow oxygen therapy at baseline remained on HFNC significantly longer than on NIV after the end of bronchoscopy, may be due to improved patient comfort under HFNC. However, this study was not designed to assess this question. Since no benefits for HFNC over NIV for bronchoscopy in hypoxaemic patients have been shown so far and HFNC therapy is, at least in our setting, associated with higher costs for disposables compared with NIV, assessment of patient comfort should also be a key element in further studies. Considering the improved oxygenation capacity of NIV in comparison with HFNC, we believe that patients with severe hypoxaemia should preferably undergo bronchoscopy using NIV. Intubation prior to bronchoscopy should be considered in patients with most severe hypoxaemia.

## Conclusions

In conclusion, the results of this study suggest, that in awake, critically ill patients with moderate to severe hypoxaemia undergoing bronchoscopy, the application of NIV was superior to HFNC regarding oxygenation before, during and after the procedure. However, in patients who were stable on HFNC, bronchoscopy was well tolerated using HFNC.

## Key messages

- Non-invasive ventilation (NIV) was superior to high-flow nasal cannula (HFNC) oxygen with regard to oxygenation before, during and after bronchoscopy in patients with moderate to severe hypoxaemia.
- In patients with stable oxygenation under HFNC, bronchoscopy was well tolerated under HFNC.

## Abbreviations

BAL: bronchoalveolar lavage
CPAP: continuous positive airway pressure
FB: flexible bronchoscopy
FiO_2_: fraction of inspired oxygen
HFNC: high-flow nasal cannula
ICU: intensive care unit
NIV: non-invasive ventilation
PaCO_2_: partial pressure of carbon dioxide in arterial blood
PaO_2_: partial pressure of oxygen in arterial blood
PEEP: positive end-expiratory pressure
SAPS II: simplified acute physiology score II
SpO_2_: oxygen saturation recorded by pulse oximetry
